# Detection of Agricultural Pesticides in Human Urine in Latvia: Links with Surrounding Land Use

**DOI:** 10.3390/toxics14010081

**Published:** 2026-01-15

**Authors:** Lāsma Akūlova, Ieva Strēle, Juris Breidaks, Anna Raita, Monta Matisāne, Linda Matisāne

**Affiliations:** 1 Laboratory of Hygiene and Occupational Diseases, Institute of Occupational Safety and Environmental Health, Rīga Stradiņš University, Dzirciema 16, LV-1007 Riga, Latvia; 2 Institute of Occupational Safety and Environmental Health, Rīga Stradiņš University, Dzirciema 16, LV-1007 Riga, Latvia

**Keywords:** human biomonitoring, pesticides, geospatial analysis, agricultural land, biomes, wetlands, residential proximity, SPECIMEn, HBM4EU, HBM4LV

## Abstract

Environmental pesticide exposure has been linked to adverse health effects, and residential proximity to agricultural land is commonly used as a proxy for exposure; however, the contribution of non-agricultural biomes remains insufficiently explored. This study examined whether the proximity and area of different biomes are associated with the detection of selected pesticides in human urine in Latvia. Urine samples were collected from 202 participants (101 adults and 101 children) within the Human Biomonitoring for Europe (HBM4EU) study during the winter and summer seasons of 2020. A suspect screening approach using liquid chromatography–high-resolution mass spectrometry (LC-HRMS) was applied and 23 pesticides were detected (8 insecticides, 12 fungicides, 2 herbicides and triclosan, an antimicrobial ingredient used in cleaning agents). Geospatial data were analysed in Quantum Geographic Information System (QGIS) to derive biome proximity and area within a 1000 m residential buffer; associations were assessed using generalized linear mixed-effects models. Agricultural land was present within 1000 m of 93.1% of residences, yet neither its distance nor area was consistently associated with pesticide detection. Boscalid was detected in 18.4% of samples and was positively associated with wetland area across seasons (*p* < 0.001), while fludioxonil (14.7%) showed weak and heterogeneous spatial associations and pirimiphos-methyl (10.2%) showed no significant patterns. Overall, pesticide exposure was substance-specific and influenced by landscape characteristics beyond agricultural proximity, highlighting the need to integrate non-agricultural biomes into human biomonitoring in low-intensity pesticide-use settings.

## 1. Introduction

Pesticides are natural or synthetic chemical substances intended for the control of pests, weeds, and disease-causing organisms in agriculture. Their use has significantly contributed to crop protection by increasing yields and improving food availability worldwide. As commercial agriculture has developed, the importance of pesticides has increased [1]. Although pesticides play an essential role in food production and health protection, their negative impact on the environment and human health must also be considered [1].

Pesticides can enter and move through the environment in several ways: (1) during application (spraying over the crop canopy with a drone, beneath the canopy, precise manual application), a small portion of aerosols is carried by the wind beyond the treated area—known as pesticide drift; (2) some pesticides evaporate from plant or soil surfaces, especially under high temperature and low humidity conditions; (3) rainfall or irrigation can cause pesticides to be washed into surface waters—rivers and lakes; (4) water-soluble pesticides can infiltrate through the soil and reach groundwater; (5) adsorption—pesticides bind to soil particles (mainly clay and organic matter), which can either reduce or, conversely, prolong pesticide mobility [2]. In the environment, pesticides are subjected to a range of physical, chemical, and biological processes that influence their movement, transformation, and potential accumulation. These processes affect both the geographic pattern of pesticide pollution and the duration and form in which these substances persist in the environment [3].

Numerous studies have confirmed that the presence of pesticides in human biological samples is significantly higher during the pesticide application season (spring and summer months) compared to winter, when chemical treatments of crops do not occur [4,5]. In the study by Ottenbros et al., which analyzed urine samples from various European countries, seasonal differences were observed, but these varied across countries and pesticide groups. Some pesticides can persist in the environment between seasons, especially those with long half-lives or strong binding to soil [4]. Teysseire et al. analyzed urine samples from children in France and found that pesticide concentrations in urine were significantly higher during periods when nearby agricultural spraying was conducted, particularly with fungicides and herbicides [5]. However, there are also studies with contrasting findings when pesticide presence has been observed more frequently during the winter season. A study conducted in France found that the time of urine sample collection influenced the presence of specific herbicide metabolites in urine (atrazine and dealkylated triazine metabolites). These compounds were detected more often when samples were collected during the autumn–winter season compared to the spring–summer season [6]. The significance of seasonality also varies between pesticides [7].

Apart from seasonality, many other factors influence pesticide distribution in the environment, including pesticide drift, weather conditions, the proximity and size of biomes, soil pH, precipitation intensity, terrain slope, and the physicochemical properties of pesticides. One of the most significant side effects of pesticide use is pesticide drift—a situation where pesticide droplets or vapors spread beyond the treated field area and settle on adjacent areas [8,9], such as residential zones, pastures, ecologically sensitive areas, or other crops for which the specific pesticide was not intended [9]. It is estimated that during agricultural spraying, approximately 30–50% of the total pesticide volume can be released into the atmosphere. This phenomenon is attributed to both mechanical dispersion (due to wind) and active volatilization [10]. Drift not only reduces the effectiveness of pesticides on target fields but also significantly increases the risk of unwanted exposure to humans, animals, and the environment [11]. Studies indicate that pesticide drift is influenced by several factors, which can be systematically divided into four groups: (1) meteorological conditions, (2) spraying equipment and method, (3) the physical properties of the spray, and (4) the operator’s skills and decisions [9]. For example, nozzle types vary, and their selection influences how fine the pesticide droplets are (the smaller the droplets, the greater the likelihood that they will be carried beyond the field boundaries) and how evenly they are applied to crops [8]. Droplets smaller than 100 μm are particularly unstable and are the most prone to dispersion [12]. In addition, operator competence is equally important—incorrect selection of spraying height or speed, use of poor-quality equipment, or choosing an inappropriate time for application, such as during strong winds, can significantly increase the risk of drift [8,12]. In practice, this means that even the best equipment can be ineffective if used carelessly or by someone insufficiently trained. Overall, pesticide drift is a complex, multifactorial process, and understanding environmental conditions and technological precision is crucial. To reduce harm to the environment and human health, technological optimization, professional training, and responsible application practices are all necessary [8,9,12].

The main determinants of exposure related to spatial factors are the distance from the residence to agricultural fields, the area of farmland surrounding the home, and the amount of pesticides used nearby. These factors are often associated with elevated pesticide concentrations in urine or household dust [5]. Significant differences between groups living near agricultural land were observed only in some countries and for specific pesticides. For example, in the Netherlands, the chlorpropham metabolite was significantly more frequently detected in agricultural areas (odds ratio (OR) = 2.1), and in Hungary, clothianidin metabolites were also more commonly found in such areas (OR = 2.8). In contrast, in Spain, the Czech Republic, and Latvia, no significant differences between exposed groups were generally observed (*p* > 0.05) [4]. Similarly, the study by Habran et al. reported that the highest pesticide concentrations and the greatest diversity were found in agricultural areas where plant protection products are used intensively [13]. Households located within 250 m of apple, peach, corn, or wheat fields were found to have significantly higher chlorpyrifos concentrations in outdoor air and on indoor surfaces compared to homes situated farther from the fields. Likewise, herbicide concentrations in homes surrounded by larger areas of corn and soybean fields within a 750 m radius were four times higher than in homes without nearby agricultural land. Studies comparing households at various distances from fields also concluded that, for example, outdoor air concentrations of certain pesticides were five times higher in homes located 30–50 m from rice fields than in those farther away [14].

The main objective of this study was to examine how various geospatial environmental factors—specifically the proximity of different biomes to the place of residence and the area covered by these biomes within the surrounding environment—influence the presence of commonly used agricultural pesticides in human urine samples in Latvia. It was hypothesised that both the proximity and area of distinct biome types surrounding the residence are significantly associated with the detection of selected pesticides in urine samples from the Latvian population. Unlike most previous studies that focus primarily on residential proximity to agricultural land, this study explicitly integrates non-agricultural biomes and pesticide-specific behaviour to assess landscape-level determinants of human exposure in a setting characterised by relatively low pesticide use intensity.

## 2. Materials and Methods

The study analyzed the association between the detection of pesticides in biological samples (urine) and the geographical location of the residence in relation to agricultural areas. Approval to involve Latvian residents in the SPECIMEn study as part of the European biomonitoring initiative HBM4EU was received from the Ethics Committee of Rīga Stradiņš University (protocol No. 6-3/3/48, 28 March 2019).

### 2.1. Study Population and Data Collection

Data on Latvian residents were obtained from the dataset that covered persons who participated in the SPECIMEn study as part of the European biomonitoring initiative HBM4EU [4]. The participants were assigned to either a control or an exposed group (individuals were defined as those living within 250 m of agricultural land where pesticides are used in crop cultivation). Two participants from each household were included—one adult (*n* = 101) and one child aged 6 to 11 years (*n* = 101): the exposed group (50 families or 100 individuals) and the control group (51 families or 102 individuals). As our preliminary analysis showed that many of the households, even in the control group, had agricultural land within a 1000 m radius of their residence, it was decided to merge both groups. Thus, the total study population consisted of 202 participants, of whom 60 (29.7%) were male and 142 (70.3%) were female. The median age of adults was 38 years (interquartile range IQR 35.0–43.0), while the median age of children was 9 years (IQR 8.0–10.0). Most households were located in Zemgale (37, 36.6%) and Kurzeme (33, 32.7%), followed by Vidzeme (30, 29.7%), with only 1 (1.0%) from Latgale.

Urine samples were collected from each participant at two time points—first in February/March 2020 (referred to as the winter season) and again in June 2020 (the summer season), allowing for an assessment of the seasonal impact on pesticide and/or metabolite levels. The residential addresses of each participant were recorded, and household location information was used later for geospatial analysis.

### 2.2. Analysis of Biological Samples

To detect pesticides and their metabolites in urine samples, a screening approach based on liquid chromatography coupled to high-resolution mass spectrometry (LC-HRMS) was used. Sample preparation, instrumental analysis, and data processing were conducted under harmonized conditions across five European laboratories (the Netherlands, Germany, France, the Czech Republic, and Spain) as part of the SPECIMEn study. Suspect database generation, mass spectrometry data analysis, and confirmation procedures were carried out centrally, supported by harmonised quality assurance and quality control measures to ensure inter-laboratory consistency. The analytical workflow included solid-phase extraction with five-fold concentration, full-scan liquid chromatography high-resolution mass spectrometry (LC-HRMS) analysis, data processing, prioritisation of putative detections, and confirmation by tandem mass spectrometry using reference standards or human liver S9 incubations. The approach is based on a centrally curated suspect database comprising several thousand known and predicted pesticide-related compounds, including parent pesticides and their metabolites, as described by Huber et al. [15]. Only biomarkers identified with high confidence (Schymanski levels 1 and 2) were considered in the present study. Additionally, the identification efforts were prioritized for halogenated and PO_3_-containing compounds due to the scope of the project and resulted in 41 pesticides or metabolites detected in the study samples. A more detailed description is published in the work of Huber et al. [15].

Unlike traditional quantitative methods, this approach enables the identification of previously unknown or rarely occurring compounds, making it particularly suitable for complex environmental and human exposure studies [15]. As the LC-HRMS suspect screening approach provides semi-quantitative signal intensities that are not directly comparable across laboratories, pesticide detection was analysed as a binary outcome (detected vs. not detected), consistent with previous HBM4EU analyses. Each sample was assigned a binary value, indicating either the presence of a specific compound above the detection limit or its absence.

### 2.3. Selection of Pesticides to Be Included in Further Geospatial Analysis

In the original study, a total of 30 pesticides were detected, some of which were tested in multiple chemical forms, resulting in 41 distinct compounds being assessed. The tested pesticides included: 2,4-D, acetamiprid, ametoctradin, boscalid, cypermethrin, cyprodinil, flonicamid, fluazifop, fludioxonil, fluopyram, flupyradifurone, fluvalinate, chlorantraniliprole, chlorpyrifos, chlorpropham, imazalil, imidacloprid, clopyralid, clothianidin, methylpyrimiphos, penconazole, pyrimethanil, propamocarb, propyzamide, tebuconazole, thiabendazole, thiacloprid, thiamethoxam, trifloxystrobin, and triclosan, which is an ingredient in cleaning agents. These chemicals were included for LC-HRMS testing because this study prioritised halogenated and PO_3_-containing compounds, along with a few other tentative annotations from previous investigations [15].

Of the 23 pesticides detected in at least one urine sample of the Latvian study participants, only eight were found in more than 10% of the analyzed samples: acetamiprid (32.8%), chlorpropham (31.6%), boscalid (18.4%), triclosan (16.2%), fludioxonil (14.7%), pyrimethanil (14.4%), imazalil (10.7%), and pirimiphos-methyl (10.2%). In the next step, the list of those eight chemicals was reviewed to exclude pesticides that were not registered in Latvia before and during the sampling time; thus, it was assumed that the exposure came from other sources (e.g., imported fruits and vegetables). For this reason, acetamiprid, pyrimethanil and imazalil were excluded. Then, another round of review was done to identify and exclude those chemicals whose intended use is not related to agricultural spraying. This process resulted in the exclusion of two other pesticides: triclosan (used as an antifungal and antibacterial additive in cosmetic products, toothpastes and soaps, etc.) and chlorpropham (used to prevent sprouting of stored potatoes in storage facilities or warehouses). Thus, further analysis was conducted only on three pesticides: boscalid, fludioxonil and pirimiphos-methyl.

### 2.4. Acquisition and Processing of Geospatial Data

Open-access and restricted-access geospatial data were obtained and combined from several national institutions and registries, including the Rural Support Service (agricultural land area and crop types), the State Forest Service registry (forest areas and classification of forest types), and the Nature Conservation Agency database (protected natural areas, microreserves, specific plant and animal species). These data provided comprehensive information on areas in Latvia with an increased likelihood of pesticide use.

Open-access data were processed using Geographic Information System (GIS) software, specifically Quantum Geographic Information System (QGIS 3.40.0 Bratislava), along with digitized maps to characterise the proximity and area of different biomes surrounding participants’ residences within a 1000 m buffer, serving as proxies for environmental exposure contexts. Proximity analysis was applied to identify zones located within 1000 m of potential high-exposure sources, such as intensively used agricultural land. No environmental samples from these biomes were collected; instead, biome characteristics were inferred from spatial land-cover data linked to residential locations. The selected biomes—agricultural land, forests, wetlands, grasslands and pastures, rivers and lakes, microreserves and coastline and dunes —were chosen based on their relevance to pesticide use, environmental fate, and potential exposure pathways in Latvia.

The initial data structure included a detailed classification of various specific biome types. To improve data clarity and analytical usability, these biome types were thematically grouped into broader categories:

- Forest types were combined into a single variable, including: alluvial forests (alluvial, riparian, and floodplain forests), mixed oak, elm, and ash forests along large rivers, herb-rich spruce forests, slope and ravine forests, oak, linden, and hornbeam forests, swamp forests, wet alder forests, old mixed broadleaf forests, old or natural boreal forests, as well as forest areas identified by the State Forest Service;
- Under the wetland biome category, the following were grouped: active and degraded raised bogs undergoing natural regeneration, great fen-sedge (*Cladium mariscus*) stands in lakes and bogs, eutrophic tall-herb communities, wet grasslands on periodically drying soils, mineral-rich springs and spring mires, and transitional bogs and fens;
- The coastal biome included foredunes, stone reefs in the sea, grey dunes with herbaceous vegetation, perennial plant communities on rocky shores, lagoons, moist interdune depressions, coastal meadows, dry heathlands on sandy coastal plains, sandy beaches with perennial vegetation, annual plant communities on muddy and low sandy beaches, wooded coastal dunes, annual plant communities on drift zones, and embryonic dunes;
- Rivers and lakes were grouped into a single variable, which included natural river stretches and rapids, lakes with characteristic aquatic vegetation, eutrophic lakes, and scenic water features such as sandstone or carbonate outcrops and undisturbed caves;
- The grassland and pasture biome category included restored EU-protected grasslands, moderately wet meadows, wooded pastures, floodplain meadows, park-like pastures and meadows, species-rich meadows, and matgrass grasslands;
- Agricultural land included a variety of crop types—vegetables, cereals, legumes, green mass crops, fodder crops, perennial plantations, oil and fiber crops, energy crops, as well as registered agricultural grasslands;
- Protected areas (microreserves) were retained as a separate variable.

Geospatial indicators were grouped into the predefined biomes. Using the obtained geospatial data, variables were created to represent the distance to specific categories of biomes. The distances to all specific biome types within a biome were aggregated, and the shortest distance among them was selected as the new value. An area of the biomes was calculated as a proportion (%) of the total area within a 1000 m radius of the residence.

### 2.5. Data Analysis

Statistical software Jamovi 2.4 and RStudio 4.4.2 were used for data analysis. Jamovi was used to obtain descriptive statistics, including the assessment of normality using the Shapiro–Wilk test. Both geospatial indicators—distance and area—did not follow a normal distribution; therefore, median and interquartile range were used for descriptive statistics.

Generalized linear mixed-effects models (GLMMs) with a binomial error distribution and logit link function were fitted using the glmmTMB function implemented through the buildmer package in R to evaluate associations between environmental factors and binary pesticide indicators. Two separate models were run for either measures of distance or area as independent variables. As the distance values were missing for biomes not being present within a 1000 m radius, these values were imputed as 1000 m in order to include all observations in the model. The initial model included all prespecified predictor variables (either distance or area of biomes listed in Table 1 and Table 2) and a random intercept for family person number (Family_ID) to account for within-family correlation. Prior to model fitting, all continuous predictor variables were standardized by centering and scaling to unit variance (z-score transformation) to facilitate model convergence and allow comparison of effect sizes across covariates.

Model selection was performed using a combined forward and backward stepwise procedure based on likelihood ratio tests (LRTs) to identify the most parsimonious set of predictors while reducing overfitting in the context of correlated spatial variables. Significance thresholds of both α = 0.10 and α = 0.05 were examined during the selection process, with predictors sequentially removed if they did not meet the specified criterion. The family-level random effect was retained in all candidate models throughout model selection. Selection proceeded hierarchically, first determining the order of terms before backward elimination. Sensitivity analyses were conducted to evaluate the robustness of model selection and parameter estimates to the choice of significance threshold (α = 0.10 vs. α = 0.05).

The final parsimonious model included only statistically significant pesticide indicators and covariates. Fixed-effect coefficients were estimated on the log-odds scale. Missing data were handled using complete-case analysis, and models exhibiting singular fits were excluded to ensure numerical stability. Statistical inference was based on Wald z-tests, with regression coefficients and their 90% confidence intervals (CIs) reported to quantify the magnitude and precision of associations between the biome and pesticide. All models were run in the full dataset and stratified by the winter or summer season.

### 2.6. Use of Artificial Intelligence

During the preparation of this manuscript/study, the authors used ChatGPT-5.1 (OpenAI, San Francisco, CA, USA) for the purposes of generating and proofreading sections of the results, discussion, conclusions and the abstract. The authors have reviewed and edited the output and take full responsibility for the content of this publication.

## 3. Results

### 3.1. Geospatial Characteristics of Residential Surroundings

Agricultural land was the most prevalent biome within a 1000 m radius of the participants’ residences, covering a median of 31.9% of the surrounding area. In addition to agricultural areas, the most commonly observed surrounding biomes were forests (64.4%), but they typically occupied only a small proportion of the area. Biomes with water: rivers and lakes were identified near 47.5% households. Microreserves, grasslands and pastures, wetlands, and coastal areas were present in less than one-third of the residential surroundings. The median distances to these biomes were generally greater than the distances to agricultural land (see Table 1).

Analyzing specific agricultural biomes, it was identified that grasslands were the most frequently present near households (around 90%), followed by cereals and “other” agricultural lands (each occurring near 80%). However, cereals were typically located closest to homes and closer than grasslands (with median distances of 137.4 m and 230.9 m, respectively) and also occupied the largest proportion of area among all agricultural biomes (around 9%). In contrast, perennial plantations, legumes and pulses, vegetables and green mass, fodder, and root crops were generally farther away and occupied much less (see Table 2).

### 3.2. Factors Associated with the Detection of Boscalid in Urine

For boscalid, several biome-related factors were associated with its detection in urine, with patterns differing by season. In the model including both seasons, a larger wetland area was positively associated with boscalid detection (regression coefficient = 0.731, *p* < 0.001), while distance to wetlands showed a borderline significant negative association (regression coefficient = −0.339, *p* = 0.057); no other variables remained statistically significant. In the winter season, the wetland area remained positively associated with boscalid detection (regression coefficient = 0.858, *p* = 0.016), whereas forest distance showed no statistically significant association. During the summer season, shorter distances to wetlands (regression coefficient = −0.519, *p* = 0.016), vegetable fields (regression coefficient = −0.501, *p* = 0.038), and coastal areas and dunes (regression coefficient = −0.467, *p* = 0.039), as well as a larger wetland area (regression coefficient = 0.769, *p* = 0.009), were all significantly associated with an increased likelihood of boscalid detection (see Table 3).

### 3.3. Factors Associated with the Detection of Fludioxonil in Urine

For fludioxonil, several biome-related factors were retained in the regression models, with associations differing by season. In the model including both seasons, a larger area of rivers and lakes was positively associated with fludioxonil detection (regression coefficient = 0.354, *p* = 0.024), while grassland and pasture area showed a negative association (regression coefficient = −0.850, *p* = 0.038); wetland area showed a borderline significant positive association (regression coefficient = 0.247, *p* = 0.075). In the winter season, fludioxonil detection was positively associated with the area of rivers and lakes (regression coefficient = 0.551, *p* = 0.025). At the same time, distance to perennial crops and forests showed borderline significant positive associations (regression coefficients = 0.570, *p* = 0.068 and 0.514, *p* = 0.072, respectively), and grassland and pasture area remained negatively associated (regression coefficient = −1.688, *p* = 0.071). During the summer season, only the wetland area remained in the final model and was positively associated with fludioxonil detection (regression coefficient = 1.474, *p* = 0.021) (Table 4).

### 3.4. Factors Associated with the Detection of Pirimiphos-Methyl in Urine

For pirimiphos-methyl, no statistically significant associations with the analysed biomes were identified. In the model including both seasons, distance to rivers and lakes showed a negative association with pirimiphos-methyl detection (regression coefficient = −0.275, *p* = 0.076), but this did not reach statistical significance. In the winter season, the vegetable area was retained in the model but was not significantly associated with pirimiphos-methyl detection (regression coefficient = −2.614, *p* = 0.142), while no biome-related variables remained in the final model for the summer season (Table 5).

## 4. Discussion

The aim of this study was to analyze whether there is an association between the proximity of a residence to different biomes—particularly agricultural land—and the detection of pesticides in human urine samples, using the Latvian population as an example. The detection of specific pesticides (boscalid, fludioxonil and pirimiphos-methyl) was examined in relation to the distance to particular landscape features and the area of these biomes surrounding the residence.

The observed associations between boscalid detection in urine and wetland-related indicators suggest that wetlands may play an active role in shaping exposure patterns rather than acting solely as protective buffers [16]. Across both seasons, a larger wetland area surrounding the residence was consistently associated with a higher risk of boscalid detection, indicating that wetlands may function as environmental reservoirs for this compound. Boscalid’s Groundwater Ubiquity Score (GUS) is 2.5, which theoretically indicates a moderate risk of leaching into groundwater [17]. Boscalid is also relatively persistent and moderately hydrophobic, and therefore prone to accumulation in moist, organic-rich environments such as wetlands, with subsequent transfer to humans via water, soil or food chains [16,18,19]. During the summer season, additional associations with shorter distances to vegetable fields and coastal areas and dunes point to a more complex exposure scenario, where local agricultural applications may interact with specific landscape features that facilitate transport and retention. Boscalid is a broad-spectrum fungicide widely used for the protection of cereals (winter and spring wheat, barley, triticale, rye and oats), oilseed crops (winter and spring oilseed rape), legumes (field beans and peas grown for grain), and a range of vegetables and fruits, including cabbages, onions, carrots, peas, garlic, plums, cherries, strawberries, cranberries and blueberries. Many of these crops are common in the Latvian agricultural landscape and are frequently cultivated in close spatial proximity to wetlands, drainage systems, and water-retaining soils, particularly in low-lying areas. Together, these findings suggest that boscalid exposure in this setting is influenced by a combination of land use and biome characteristics, underscoring the importance of considering non-agricultural biomes when assessing landscape-level determinants of pesticide exposure.

In contrast to boscalid, the spatial patterns observed for fludioxonil were less consistent and more season-dependent, suggesting a weaker and less stable relationship with surrounding land-use characteristics. While positive associations were identified with the area of rivers and lakes across both seasons and during the winter season, and with wetland area during the summer season, these effects were not uniform and were accompanied by negative associations with grasslands and pastures. The lack of consistent distance-based effects and the variability across seasons indicate that residential proximity to specific biomes may only partially capture fludioxonil exposure pathways. Fludioxonil is a broad-spectrum fungicide used across a wide range of crops, including cereals (winter and spring wheat, barley, rye, triticale and oats), potatoes, strawberries, raspberries, blackberries, vegetables grown both in open fields and protected cultivation (e.g., tomatoes, cucumbers, peppers, aubergines, courgettes, pumpkins, salads, carrots, onions and garlic), as well as ornamental plants and woody species in nurseries. This highly diverse crop-use profile, spanning open-field agriculture, covered production systems and non-food plant cultivation, likely contributes to the weak and heterogeneous spatial associations observed in the present study. Unlike pesticides predominantly applied in uniform open-field settings, fludioxonil applications may occur in spatially fragmented and partially enclosed environments, where direct environmental dispersion is reduced and exposure pathways are less strongly linked to residential proximity or simple land-use indicators. Given fludioxonil’s low water solubility and strong sorption to soil and organic matter, the observed associations with rivers, lakes and wetlands may therefore reflect indirect transport processes, such as runoff or drainage from treated areas or or dietary intake, rather than direct drift from nearby agricultural fields [19,20,21]. Overall, the results suggest that fludioxonil exposure in this setting is not strongly structured by simple spatial indicators, highlighting the need for integrating land-use data with pesticide-specific properties and individual exposure pathways when interpreting human biomonitoring data.

For pirimiphos-methyl, no clear or statistically robust associations with surrounding biomes were identified, suggesting that residential land-use characteristics played a limited role in shaping exposure patterns in this setting. The absence of consistent spatial or seasonal effects may be partly explained by the primary use pattern of pirimiphos-methyl, which is commonly applied as an insecticide for stored grain and indoor pest control rather than for open-field spraying [22]. Consequently, exposure pathways are likely dominated by indirect sources, such as dietary intake of treated commodities or indoor use, rather than by proximity to agricultural land or specific landscape features. The borderline association observed for distance to rivers and lakes in the combined model should therefore be interpreted cautiously and may reflect residual confounding or chance rather than a meaningful environmental transport pathway. Overall, these findings indicate that pirimiphos-methyl exposure is weakly structured by geospatial indicators, underscoring the importance of accounting for pesticide-specific use patterns and non-environmental exposure routes when interpreting biomonitoring data.

In several countries, the literature indicates that proximity to intensive agricultural areas is associated with higher levels of pesticide detection in biological samples [5,14]. However, the present findings only partially support this relationship. In this Latvian population, neither the distance to nor the area of agricultural land showed a consistent association with pesticide detection across the three analysed compounds, suggesting that agricultural proximity alone is an insufficient proxy for exposure. This divergence from the international literature may be partly explained by the comparatively low intensity of pesticide use in Latvia, where application rates are substantially lower than the EU average, resulting in limited exposure contrasts between residential settings. According to the State Plant Protection Service, in 2019, the use of plant protection products in Latvia was 0.84 kg per hectare of agricultural land, which is less than half the EU average of 2.05 kg/ha. Despite an 8.2% increase in the total area of agricultural land and a 13.9% increase in arable land in Latvia between 2011 and 2019, the volume of plant protection product use remains one of the lowest in the EU [23]. Under such conditions, pesticide-specific use patterns, physicochemical properties, and interactions with surrounding landscape features appear to play a more important role in shaping biomonitoring outcomes than proximity to agricultural land per se.

It is also worth noting that the literature highlights the protective function of certain biomes. Forests, wetlands, and other ecosystems have been mentioned as potential buffers that can reduce pesticide drift and exposure [24]. In our study, however, the findings do not provide consistent support for this buffering effect. Forests and grasslands were generally not associated with a reduced likelihood of pesticide detection for any of the analysed compounds, and for boscalid, larger wetland areas were in fact associated with a higher probability of detection, suggesting a reservoir or retention function rather than a protective one. For fludioxonil, associations with water-related biomes were heterogeneous and directionally inconsistent, while pirimiphos-methyl showed no clear spatial pattern, further indicating that natural biomes did not systematically mitigate exposure in this setting. Together, these results suggest that the role of biomes as protective buffers is highly context- and substance-dependent and may be outweighed by pesticide-specific properties, use patterns, and local environmental conditions.

Seasonality is repeatedly recognized in the literature as one of the strongest determinants of exposure [4,5,6,7]. However, in the present study, seasonal variation in pesticide detection was less pronounced than expected and appeared to be partly outweighed by landscape-specific characteristics. No statistically significant differences were observed between the summer and winter seasons in overall pesticide detection or for fludioxonil and pirimiphos-methyl, while for boscalid, seasonality influenced the relevance of specific spatial predictors rather than the overall likelihood of detection. Several factors may explain these findings. Urine samples were collected at only two fixed time points, which may not have coincided with short and variable pesticide application periods, leading to potential exposure misclassification. This is particularly relevant in Latvia, where climatic conditions and application practices may result in spraying occurring outside the predefined sampling windows, and exposure may take place during brief, unpredictable time intervals that short-term biomarkers cannot readily capture [5]. In addition, the relatively low and heterogeneous intensity of pesticide use in Latvia may reduce contrasts between summer and winter seasons, while cooler temperatures, higher humidity, and variable wind patterns may further limit volatilization and long-range drift, contributing to more diffuse and less seasonally distinct exposure patterns.

The strengths of this study include several key aspects that enhance the reliability and applicability of the results. Despite the limited number of measurements, the study design included seasonal repeated sampling from the same individuals, both during and outside of the spraying season. This allowed for partial control of inter-individual variation and provided an opportunity to assess seasonal changes at the individual level. Particular attention was given to spatial exposure assessment, using both distance and area indicators within various buffer zones, based on official QGIS datasets. Generalized mixed models were employed in the analysis, which are suitable for data structures involving repeated measures and clustering at the family level. Data collection from several regions in Latvia increases the generalizability of the results across different environmental contexts.

This study also has several limitations that should be considered when interpreting the results. Although the dataset included 101 families with two samples per family (one during the summer season and one outside of it), the overall sample size is not large enough to fully detect all potential associations, especially when multiple independent variables are analyzed simultaneously. This study did not include detailed data on individual behavior and lifestyle factors, such as diet, occupational exposure, or pesticide use at home, which could serve as alternative explanations for the detection of pesticides in urine. This limits the ability to clearly distinguish environmental influences from other potential sources of exposure. While the detected pesticides indicate the presence of environmental exposure pathways, their detection alone does not imply adverse health effects. Because this study relied on detection frequency rather than quantified internal concentrations, it does not allow for assessment of dose–response relationships or health risk characterization.

The literature strongly emphasizes the importance of pesticide drift, noting that these substances can sometimes reach environmental samples even several kilometers away from the treated fields [24]. It should be acknowledged that this study focused solely on geospatial indicators—area and distance to the biome. This approach does not account for other environmental factors that could influence actual pesticide drift, such as meteorological indicators like wind direction, speed, precipitation, relative humidity or air temperature; terrain or physical barriers between the household and the field [12]. Wind patterns, relative humidity, and air temperature are key drivers of pesticide drift. Greater wind variability, rising temperatures, and low humidity promote more active volatilization and broader spread of pesticide vapors. It has been described in the literature that when interpreting study results, it is also important to consider that the unpredictability of environmental conditions significantly complicates the ability to conduct objective and comparative drift assessments [8]. However, we did not have access to such data. In addition, we did not have any information on the type and process of pesticide application (e.g., techniques used, equipment, training of workers performing the application, etc.) [8].

To enhance future research on pesticide exposure, it is recommended that additional variables be incorporated to capture individual exposure pathways, including dietary habits, occupational activities, and personal pesticide use, to better distinguish between potential exposure sources. Long-term human biomonitoring strategies with multiple sampling points throughout the year are also encouraged, as they would provide a more accurate assessment of seasonal variations. Furthermore, geospatial analyses should extend beyond a 1000 m radius and consider key environmental factors, such as wind direction and terrain features, that may significantly influence the drift and distribution of pesticides.

## 5. Conclusions

This study shows that residential proximity to agricultural land alone does not adequately explain pesticide detection in human urine in Latvia, highlighting the limitations of using simple distance-based indicators as proxies for exposure. Instead, exposure patterns were substance-specific and shaped by interactions between pesticide properties, use patterns, and surrounding landscape features, with wetlands emerging as a key determinant for boscalid, heterogeneous and weak spatial associations observed for fludioxonil, and no robust geospatial patterns identified for pirimiphos-methyl. Seasonality, although widely recognised as a major determinant of pesticide exposure, played a limited role in this setting, likely reflecting low and heterogeneous pesticide use intensity, climatic conditions, and the constraints of short-term biomonitoring. Overall, these findings underscore the need for human biomonitoring approaches that integrate pesticide-specific behaviour, non-agricultural biomes, and contextual environmental factors, particularly in regions with comparatively low pesticide use.

## Figures and Tables

**Table 1:** Biomes near the residences of the study population.

Biomes	Number (%) Within a 1000 m Radius (*n* = 101)	Distance ^1^ to Biomes in Meters, Median (IQR)	Area Within a 1000 m Radius, % (IQR) (*n* = 101)
Agricultural land	94 (93.1)	81.9 (31.8–325.1)	31.9 (7.1–59.6)
Forests	65 (64.4)	335.1 (56.4–6743.8)	1.0 (0.0–15.6)
Rivers and lakes	48 (47.5)	351.3 (180.0–656.1)	0.0 (0.0–0.4)
Microreserves	41 (40.6)	576.5 (287.8–779.3)	0.0 (0.0–8.0)
Grasslands and pastures	30 (29.7)	492.6 (341.7–726.1)	0.0 (0.0–0.1)
Wetlands	22 (21.8)	583.0 (371.7–850.5)	0.0 (0.0–0.0)
Coastline and dunes	13 (12.9)	239.1 (148.7–352.7)	0.0 (0.0–0.0)

^1^ Distance refers to the shortest distance from the residence to the nearest area of a given biome among the residences where the given biome is present within a 1000 m area.

**Table 2:** Agricultural biomes near the residences of the study population.

Types of Agricultural Lands	Number (%) Within a 1000 m Radius (*n* = 101)	Distance ^1^ to Biomes in Meters, Median (IQR)	Area Within a 1000 m Radius, % (IQR) (*n* = 101)
Grasslands	90 (89.1)	230.9 (112.2–534.4)	4.0 (1.1–9.3)
Cereals	80 (79.2)	137.4 (63.2–440.3)	9.3 (0.8–34.4)
Oil and fiber crops	59 (58.4)	256.6 (76.1–563.9)	1.8 (0.0–8.8)
Vegetables	53 (52.5)	480.0 (194.9–767.0)	0.0 (0.0–0.4)
Perennial plantations	49 (48.5)	561.2 (304.1–758.2)	0.0 (0.0–0.4)
Legumes and pulses	47 (46.5)	459.6 (180.0–773.5)	0.0 (0.0–2.0)
Green mass, fodder, and root crops	15 (14.9)	480.8 (417.5–665.1)	0.0 (0.0–0.0)
Other	80 (79.2)	307.3 (134.6–618.4)	0.8 (0.1–2.2)

^1^ Distance refers to the shortest distance from the residence to the nearest area of a given biome among the residences where the given biome is present within a 1000 m area.

**Table 3:** Regression analysis results on factors (biomes) associated with the detection of boscalid in urine.

Type of Land	Variable	Both Seasons				Winter Season				Summer Season			
		Regression Coefficient	*p*	90% CI		Regression Coefficient	*p*	90% CI		Regression Coefficient	*p*	90% CI	
				Lower	Higher			Lower	Higher			Lower	Higher
Wetlands	Distance	−0.339	0.057	−0.632	−0.046	a	a	a	a	−0.519	0.016	−0.873	−0.164
Wetlands	Area	0.731	<0.001	0.374	1.089	0.858	0.016	0.274	1.441	0.769	0.009	0.284	1.254
Vegetables	Distance	a	a	a	a	a	a	a	a	−0.501	0.038	−0.898	−0.104
Coastal areas and dunes	Distance	a	a	a	a	a	a	a	a	−0.467	0.039	−0.840	−0.095
Forest	Area	a	a	a	a	−0.453	0.110	−0.919	0.013	a	a	a	a
Grasslands and pastures	Area	−0.416	0.103	−0.836	0.004	a	a	a	a	a	a	a	a

a—Variable was removed during both forward and backward stepwise regression procedure.

**Table 4:** Regression analysis results on the factors (biomes) associated with the detection of fludioxonil in urine.

Type of Land	Variable	Both Seasons				Winter Season				Summer Season			
		Regression Coefficient	*p*	90% CI		Regression Coefficient	*p*	90% CI		Regression Coefficient	*p*	90% CI	
				Lower	Higher			Lower	Higher			Lower	Higher
Perennial crops	Distance	a	a	a	a	0.570	0.068	0.056	1.084	a	a	a	a
Perennial crops	Area	a	a	a	a	−0.612	0.165	−1.336	0.113	a	a	a	a
Forest	Distance	a	a	a	a	0.514	0.072	0.045	0.983	a	a	a	a
Grasslands and pastures	Area	−0.850	0.038	−1.524	−0.176	−1.688	0.071	−3.226	−0.150	a	a	a	a
Rivers and lakes	Area	0.354	0.024	0.095	0.613	0.551	0.025	0.146	0.956	a	a	a	a
Wetlands	Area	0.247	0.075	0.019	0.475	a	a	a	a	1.474	0.021	0.552	2.396

a—Variable was removed during both forward and backward stepwise regression procedure.

**Table 5:** Regression analysis results on the factors (biomes) associated with the detection of pirimiphos-methyl in urine.

Type of Land	Variable	Both Seasons				Winter Season				Summer Season			
		Regression Coefficient	*p*	90% CI		Regression Coefficient	*p*	90% CI		Regression Coefficient	*p*	90% CI	
				Lower	Higher			Lower	Higher			Lower	Higher
Rivers and lakes	Distance	−0.275	0.076	−0.530	−0.020	a	a	a	a	a	a	a	a
Vegetables	Area	a	a	a	a	−2.614	0.142	−5.545	0.317	a	a	a	a

a—Variable was removed during both forward and backward stepwise regression procedure.

## Data Availability

The data presented in this study are not publicly available due to ethical and data protection restrictions, as they contain potentially identifiable human biomonitoring and geolocation information. Anonymized data may be made available from the corresponding author upon reasonable request and with appropriate ethical approval.
